# Density scaling artifacts in dosimetry calculations

**DOI:** 10.1120/jacmp.v6i3.2095

**Published:** 2005-08-17

**Authors:** Peter Dickof

**Affiliations:** ^1^ Department of Medical Physics Allan Blair Cancer Centre 4101 Dewdney Avenue Regina Saskatchewan S4S 1V4 Canada

**Keywords:** density scaling, artifact, Pinnacle3, collapsed‐cone convolution

## Abstract

Quality assurance dosimetry often requires the comparison of measured doses with those calculated by a treatment‐planning system for phantoms of density other than 1.000 g/cm^3^. The presence of an artifact in the Pinnacle3 treatment‐planning system can lead to systematic errors in such cases.^a^ These errors are also present, although reduced in magnitude, in heterogeneous media.

PACS: 87.53.Bn, 87.53.Xd

## I. INTRODUCTION

Film dosimetry is often done with the intent of comparing the dose calculated by a treatment‐planning system with that measured in a solid phantom. Since the commercially available solid phantoms have densities other than $1.000\;g/{\text{cm}}^{3}$, the performance of the planning system in a uniform phantom is a key parameter. In this article, we demonstrate the existence of artifacts in a widely used treatment‐planning system. We also estimate the size of these artifacts in a heterogeneous patient pelvis.

## II. METHODS

The Pinnacle3 v6.2b treatment‐planning system was used to generate phantoms of uniform density by defining a 30×30×20 cm region of interest and modifying the density of this region. The outside‐patient air threshold was changed when required to calculate dose to low‐density phantoms. Points were defined at depths as required, and the dose to a point was calculated using the collapsed‐cone convolution algorithm for a prescription of 10^5^ monitor units.

## III. RESULTS AND DISCUSSION

**Figure 1:**
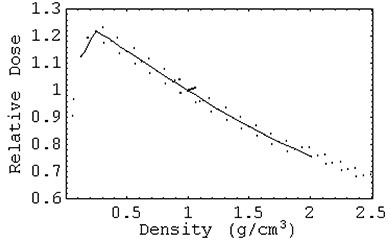
The Pinnacle3‐calculated dose (normalized to that for unit density) at a depth of 10 cm for a 6‐MV 10×10 cm field incident at 90 cm SSD on a phantom of variable density. The individual calculation densities are shown along with a line joining the center of each linear range.

Figure 1 shows the normalized dose calculated by Pinnacle for a 10×10 cm 6‐MV field incident on the phantom at 90.0 cm source‐to‐detector distance (SSD) to a point 10.0 cm deep as the density of the phantom is changed in the range $0.06\;g/{\text{cm}}^{3}$ to $6.76\;g/{\text{cm}}^{3}$. Doses are normalized to that for density $1.00\;g/{\text{cm}}^{3}$.

A marked step pattern is present with sharp decreases in dose at densities of approximately $(i/8‐1/16)g/{\text{cm}}^{3}$ for i=1,15, and $(2+(i‐16)/8)g/{\text{cm}}^{3}$ for *i* thereafter. There is an apparent shift in the step pattern near $2\;g/{\text{cm}}^{3}$. Discussion with the manufacturer indicated that the step pattern was due to a nearest‐neighbor interpolation into a table of linear attenuation coefficients during the primary ray‐trace. This nearest‐neighbor binning introduces density errors that are maximized when the density is homogeneous and lies near one of the transition points. The shift in the step pattern near $2\;g/{\text{cm}}^{3}$ is not currently understood.

The variation in size of the dose steps as a function of depth and energy is shown in Table 1. Dose calculations were done for a 10×10 cm beam incident at 90 cm SSD on a phantom with densities of $1.0627\;g/{\text{cm}}^{3}$ and $1.0628\;g/{\text{cm}}^{3}$ at each of the depths and energies specified. The percentage differences shown in Table 1 were calculated as follows:

(1) $$dose\;Difference=\frac{\left({dose}_{1.0627}-{dose}_{1.0628}\right)}{({dose}_{1.0627}+{dose}_{1.0628})/2}\times 100\%$$

**Table 1:** Percentage dose difference between phantom densities of $1.0627\;g/{\text{cm}}^{3}$ and $1.0628\;g/{\text{cm}}^{3}$ calculated at various depths by Pinnacle3 v6.2 for 10×10 cm beams of various energies incident on the phantom at 90 cm SSD

Depth		Energy	
	6 MV	10 MV	16 MV
5 cm	3.0%	2.2%	1.8%
10 cm	5.3%	4.0%	3.5%
15 cm	7.6%	5.9%	5.2%

Essentially no variation in the size of the dose steps was seen with a change in field size to 20 cm or a change in the resolution of the Pinnacle3 calculation grid.

The manufacturer has told us that the Pinnacle3 software creates a mass attenuation coefficient lookup table at the beginning of a dose calculation. This table stores mass attenuation coefficients for the selected beam as a function of

1. density to account for different materials; the coefficients are determined by first‐order (i.e., linear) interpolation of the data found in usr\local\adacnew\PinnacleStatic_6.2b\PhysicsData\MassAttenuationTables.db
2. radiological depth to account for beam hardening
3. off‐axis angle to account for off‐axis softening

When ray tracing is done, interpolation from this table is zeroth‐order (i.e., nearest‐neighbor). Scatter calculation is done by ray tracing and density‐scaling water scatter kernels via first‐order interpolation. The material to which the dose is calculated is always that entered when the absolute output is entered into Pinnacle3 (typically muscle or water). The observed steps are due to the nearest‐neighbor density lookup in the primary ray‐trace. In version 6.2, the density bins have a width of $1/8g/{\text{cm}}^{3}$ the bin width is reduced to $1/16g/{\text{cm}}^{3}$ in version 7.4.

We have estimated correction factors for both version 6.2 and 7.4 algorithms at a depth of 10 cm for the density of a few materials commonly used for dosimetry as follows:

(2) $$correctionFactor=1-\frac{physicalDensity-pinnacleDensity}{pinnacleDensityBinWidth}\times \frac{doseDifference}{100},$$

where doseDifference is taken from Table 1, pinnacleDensityBinWidth is the width of the density used by each version of Pinnacle, and pinnacleDensity is the density value used by each version of Pinnacle as a result of binning. The correction factors to apply to Pinnacle3 doses to return true dose are given in Table 2. Note that the change to the v7.4 algorithm results in errors that are either unchanged or are changed in sign and reduced.

**Table 2:** Estimated correction factors applicable to doses calculated by the Pinnacle3 planning system at 10 cm depth for different densities. In each case, the first number is for version 6.2, the second for version 7.4.

Material (density)		Energy	
	6 MV	10 MV	16 MV
water (ρ=1.000)	1.000/1.000	1.000/1.000	1.000/1.000
solid water (ρ=1.014)	0.993/0.993	0.995/0.995	0.996/0.996
polystyrene (ρ=1.04)	0.982/1.011	0.987/1.008	0.989/1.007
acrylic (ρ=1.17)	0.980/1.008	0.985/1.006	0.987/1.005

**Figure 2:**
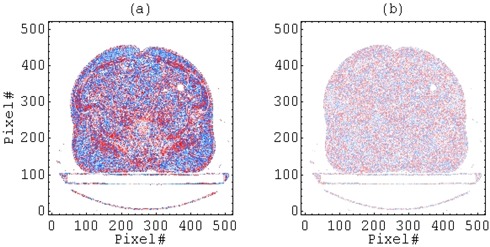
The density error introduced by Pinnacle3 due to binning. Color indicates sign: the density estimate is too high when red, too low when blue. Color saturation indicates magnitude; full saturation is achieved at an error magnitude of $1/16g/{\text{cm}}^{3}$. Density bin sizes are (a) $1/8g/{\text{cm}}^{3}$ (v6.2) and (b) $1/16g/{\text{cm}}^{3}$ (v7.4).

We have written a computer program to estimate the size of the errors in a patient pelvis CT scan. All pixels were converted from Hounsfield units to density using the data in our CT density tables. The density error in each was obtained by subtracting the true density from the density of the binned value. The density errors are shown in Figure 2; there is clear structure in the errors and a clear preponderance of negative density errors when the bin size is $1/8g/{\text{cm}}^{3}$ ((Fig. 2a). Cumulative sums of the density errors in a row of pixels allow us to estimate the cumulative density error for a lateral beam; comparing this with the cumulative density errors in the homogeneous cases allows us to interpolate estimated dose errors. A dose error distribution of 0.3±0.4% is obtained on the patient midplane. Changing from the v6.2 to the v7.4 algorithm halves the mean density error per pixel and distributes these errors more symmetrically about 0, resulting in an improved dose error distribution of –0.04±0.1% on the patient midplane.

## IV. CONCLUSION

The data presented show an artifact in the Pinnacle3 collapsed‐cone convolution dose calculation engine that results in steps in the dose calculated as a function of density. The steps occur at fixed density positions and imply a systematic error in the calculated dose at any given density. The shift at $2\;g/{\text{cm}}^{3}$ implies an additional systematic error for densities above this value. Such systematic errors could be significant for quality assurance calculations done for nonunit‐density phantom materials. Their effects on heterogeneous phantoms tend to be smaller due to partial cancellation.

